# Nitrogen fertilization impact on soil carbon pools and their stratification and lability in subtropical wheat-mungbean-rice agroecosystems

**DOI:** 10.1371/journal.pone.0256397

**Published:** 2021-10-01

**Authors:** Rafeza Begum, Mohammad Mofizur Rahman Jahangir, M. Jahiruddin, Md. Rafiqul Islam, Md. Taiabur Rahman, Md. Lutfar Rahman, Md. Younus Ali, Md. Baktear Hossain, Khandakar Rafiq Islam

**Affiliations:** 1 Department of Soil Science, Bangladesh Agricultural University, Mymensingh, Bangladesh; 2 Soil Resource Development Institute, Jamalpur, Bangladesh; 3 Soil Resource Development Institute, Dhaka, Bangladesh; 4 SAARC Agriculture Center (SAC), Dhaka, Bangladesh; 5 The Ohio State University, Columbus, Ohio, United States of America; Government College University Faisalabad, PAKISTAN

## Abstract

Nitrogen (N) is the prime nutrient for crop production and carbon-based functions associated with soil quality. The objective of our study (2012 to 2019) was to evaluate the impact of variable rates of N fertilization on soil organic carbon (C) pools and their stocks, stratification, and lability in subtropical wheat (*Triticum aestivum*)—mungbean (*Vigna radiata*)—rice (*Oryza sativa* L) agroecosystems. The field experiment was conducted in a randomized complete block design (RCB) with N fertilization at 60, 80, 100, 120, and 140% of the recommended rates of wheat (100 kg/ha), mungbean (20 kg/ha), and rice (80 kg/ha), respectively. Composite soils were collected at 0–15 and 15–30 cm depths from each replicated plot and analyzed for microbial biomass (MBC), basal respiration (BR), total organic C (TOC), particulate organic C (POC), permanganate oxidizable C (POXC), carbon lability indices, and stratification. N fertilization (120 and 140%) significantly increased the POC at both depths; however, the effect was more pronounced in the surface layer. Moreover, N fertilization (at 120% and 140%) significantly increased the TOC and labile C pools when compared to the control (100%) and the lower rates (60 and 80%). N fertilization significantly increased MBC, C pool (CPI), lability (CL_I_), and management indices (CMI), indicating improved and efficient soil biological activities in such systems. The MBC and POC stocks were significantly higher with higher rates of N fertilization (120% and 140%) than the control. Likewise, higher rates of N fertilization significantly increased the stocks of labile C pools. Equally, the stratification values for POC, MBC, and POXC show evidence of improved soil quality because of optimum N fertilization (120–140%) to maintain and/or improve soil quality under rice-based systems in subtropical climates.

## Introduction

Subtropical agricultural soils are under intense pressure to support food security in response to increasing global population growth and climate-change effects. These soils are generally N deficient under hot and humid conditions and require N optimization to support crop production and to maintain or improve soil quality. Management practices that optimize N fertilization for crops can improve N-use efficiency, maintain soil N level, or deliver N to the environment when excessive N is applied [1]. Post-applied N fertilization is often taken up by the crops or circulated within the soil-water-air environments [2]; however, excessive N fertilization could be associated with reduced harvest index and emission of greenhouse gases, especially nitrous oxides, with increased ecosystem disservices. Jeppsson [3] has reported that excessive N fertilization reduces crop yield and affects the composition and quality of produce. Excessive N fertilization is often associated with groundwater’s nitrate pollution and public health problems like methemoglobinemia [4]. Likewise, inadequate supply of N fertilizer can reduce soil native N stock, causing progressive N deficiency.

While C and N are stoichiometrically linked in soil organic matter (SOM) to maintain functional stability of the terrestrial ecosystems [5], excessive or reduced N fertilization over time is expected to impact TOC content and quality or vice-versa. Previous studies have reported variable impacts (positive, neutral, or negative) of N fertilization on TOC dynamics under diverse management practices [6–10]. While it is reported that N fertilizer increases crop yield and biomass production, and thus, increases TOC content, others reported a decrease in TOC content by accelerating mineralization of native SOM [11]. However, an absolute change in bulk TOC content often takes several years to detect by management practices.

There were studies that emphasize the labile C pool, a fraction of TOC, that are sensitive and can provide early indications of management-induced changes [12–14]. Several TOC pools and C-based quotients that are considered as labile are POC), dissolved organic C, MBC, active C (AC) or POXC, potentially mineralizable C (PMC), light C fraction (LF), hot-water soluble C (HWSC), anthrone reactive C, metabolic quotient (qR), BR, specific maintenance respiration (qCO_2_), and CMI, which have been suggested as sensitive indicators of TOC change in response to management practices [15–19].

While several studies reported that impact N fertilization influences soil microbial populations and their respiratory and enzymatic activities [20], there is a lack of available information on the impact of N fertilization on soil labile C pools as influenced by cereal-legume-based cropping systems under subtropical soil and climatic conditions. Our hypothesis is that N fertilizer management increases the concentration and stocks of biological, chemical and physical C pools associated with the TOC lability under a diverse cropping sequence in subtropical agroecosystems. Thus, the objective of our study was to evaluate the long-term effects (2012–2019) of N fertilization on the concentration of TOC, biological, chemical, and physical C pools and their stocks, stratification, and lability at different depths in a wheat-mungbean-rice cropping sequence under subtropical climatic conditions.

## Materials and methods

### Experimental site

The study was conducted on the Bangladesh Agricultural University experimental farm, (24° 43.407ʹ N, 90° 26.22ʹ E), beginning in 2012 under a wheat-mungbean-rice cropping sequence. The climate is subtropical monsoon with a mean annual temperature of 26°C, average annual rainfall of 180 cm, and relative humidity ranging from 65 to 96%. Soil is a non-calcareous dark grey floodplain soil (Old Brahmaputra Floodplain soil), which is classified as Inceptisols, having silt loam texture [21].

### Experimental design and cultural practices

The wheat-mungbean-rice cropping sequence experiment was established in a RCB design with N fertilization at 60, 80, 100, 120, and 140% of the recommended doses. Here, 100% N-recommended dose was used as the control. The 100% recommended rates of N fertilization were 100, 20, and 80 kg/ha for wheat, mungbean and rice, respectively. Each replicated plot was 7 m long x 3.5 m wide with a 50-cm buffer between plots. The field preparation was initiated during the third week of November 2012 for planting wheat, followed by planting of mungbean in the last week of March 2013, and the transplanting of aman rice seedlings in the last week of July 2013. While the seeding rates were 120 and 30 kg/ha for wheat (cv. BARI Gom-30) and mungbean (BINA mung-8), respectively, the rice (BRRI dhan-9) transplanting rates were 250,000 seedlings/ha with 20 cm between seedlings and 20 cm between rows. Wheat season was from the last week of November 2012 to mid-March 2013 (Rabi season), followed by mungbean from early April 2013 to late June 2013 (pre-monsoon/pre-kharif season), and rice as a rainfed crop from early July 2013 to November 2013 (monsoon/kharif season). The cropping sequence was repeated over a period of seven years until the end of 2019.

N was applied in three equal splits at 0, 25, and 50 days after planting wheat, as a single starter application for mungbean, and as three splits at 10 (50% at seedling stage), 30 (25% at tillering stage), and 50 days after transplanting (25% at panicle initiation stage) for rice. Other nutrients, namely P, K, S, Zn, and B, were applied at 20, 60, 10, 2, and 1.5 kg/ha, respectively, for wheat; 20 kg P, 30 kg K, and 10 kg S/ha for mungbean; and 10 kg P, 30 kg K, 10 kg S, and 2 kg Zn/ha for rice.

Nonselective herbicide glyphosate (Roundup^®^; Bayer Bangladesh.) was applied at 1.85 kg a.i./ha at 2 to 3 days before transplanting of rice seedlings and planting of wheat and mungbean. In addition, Pretilachlor (Superhit^®^, post emergence herbicide; ACI Bangladesh Ltd.) was applied at 450 g a.i./ha at 5 to 7 days after transplanting rice seedlings. Insecticide Brifar 5G (ACI Bangladesh Ltd.) was applied 50 days after planting wheat, Diazinon was sprayed three times for mungbean (36, 48, and 59 days after planting, respectively) and insecticides Brifer 5G and Cidial 5G were applied to control insects for rice. Supplemental irrigation was provided twice for wheat and once during the crown roots initiation, then again at the flowering stage. The rice fields were irrigated one day before the final land preparation (soil puddling).

### Soil sampling and analysis

Soil samples were randomly collected from two soil depths (0-15cm and 15-30cm) from each of the three replicated plot using a 10 cm internal diameter auger. From each replicated plot several single soil samples were collected and a total of 30 composite soil samples was prepared. A portion of field-moist soil was processed after sieving through a 2-mm mesh to remove visible organic residues and rock pieces (if any), then analyzed for MBC and associated biological properties. The other portion of the field-moist soil was air-dried under shade at room temperature (~25 °C) for two weeks and processed (2 mm sieved) to analyze for selected physical and chemical properties.

The MBC, as a measure of biological C pool, was determined by chloroform (CHCl_3_) fumigation extraction method [22]

MBC(mg/kg)=EC/kEC

Where the EC is the flash of extracted organic C from CHCl_3_ fumigated soil minus organic C extracted from non-fumigated soil, and kEC is the extraction efficiency coefficient (0.45).

The BR, as a measure of soil biological activity, was measured by following the *in-vitro* steady static incubation method [23]. Likewise, the PMC was determined using the in-vitro static incubation of field-moist soil [24]. Briefly, a 20-g sample of processed field-moist soil was taken in 25-ml glass beakers and adjusted at 70% water-filled porosity for incubation in 1-L mason jars along with a glass vial containing 10 mL of distilled water to maintain a moist atmosphere and a plastic vial containing 20 mL of 0.5-M NaOH for absorption of emitted CO_2_. The mason jars were sealed airtight and incubated in the dark at 25+1°C for a 10-day period. The CO_2_ released from the soil during incubation was absorbed by the 0.5-M NaOH and was titrated against the standard 0.5-M HCl solution to calculate for PMC pool after diving with the TOC concentration. The qCO_2_, as a measure of C used for microbial catabolism, were calculated by dividing the BR rates with the MBC concentration [24, 25].

The TOC concentration was determined by following the standard wet oxidation method [26]. The POXC, as a measure of chemically labile C pool, was determined spectrophotometrically upon mild oxidation of air-dried soil with a neutral KMnO_4_ solution [27]. The POC was determined after extracting particulate organic matter (POM) following the method described by Cambardella and Elliott [28]. The POM was separated from soil by adding 240 mL of 0.5% Na-hexametaphosphate solution to 80 g of air-dried soil, followed by shaking for 18 hr. and then wet sieving the soil slurry through a 53-μm mesh under running water [28]. Sand particles and POM remaining on the sieve were then oven-dried at 105°C for 30 min then weighed, burned in a muffle furnace at 460°C for 16 h, and weighed again in a manner consistent with the loss of ignition procedure. Soil bulk density (ρb) was determined by following the standard core method [29]. Antecedent soil moisture content was measured using the thermo-gravimetric method [26].

### Soil carbon stocks and stratification

Soil organic C stocks at different depths were calculated by multiplying their concentration with the sampling depth interval and concurrently measured antecedent ρb. The stratification of C pools was calculated by dividing their concentration at each depth with the concentration of the respective C pools at the deeper depth (15–30 cm) under the control [30]. The use of control soil lower depth to calculate for C stratification is important, so that the impact of N fertilization under similar soil and climate conditions can be compared and contrasted.

### Soil carbon lability and management indices

Using the measured TOC, MBC, POC, and POXC data, the CMI was calculated [19, 31] as follows:

CMI=CPI×CLI

where CPI is the C pool index and CL_I_ is the C lability index, which were calculated as:

CPI = TOC in the treatment soil/TOC in the control soil under CT

CL_I_ = CL in the treatment soil/CL in the control soil under CT

where CL refers to the lability of C (CL = Labile C/Non-labile C).

The labile C pool was considered the portion of TOC that was measured as the POXC pool. The non-labile C pool was calculated by subtracting the POXC content from the TOC. The calculated CMIs were normalized (CMI) by dividing the values with the highest CMI values in the database to a relative scale of >0 to <100, considering higher CMI values are better indicators of TOC accumulation and lability in response to N management practices.

### Statistical analysis

A two-way analysis of variance (ANOVA) was performed using N rates and depth as fixed variables and block as a random variable. The distribution of data for normality was checked before ANOVA. Data were statistically analyzed to ascertain the significant differences in simple effects and interaction of N x depth using SAS^®^ [32]. A post-hoc test was performed to separate differences between N rates using the Tukey-Kamer’s multiple comparison. All statistical analyses were considered significant at p<0.05, unless otherwise mentioned. Regression and correlation analyses were performed using SigmaPlot^®^.

## Results

### Nitrogen fertilization impact on soil organic carbon pools

Nitrogen fertilization significantly affected the soil biological C pools and processes of MBC, BR, and qCO_2_ (Table 1). Averaged across depth, the MBC was significantly higher by 18% when N was applied at 120 and 140% than that at 60%, where the former two rates were statistically alike. No significant differences in MBC contents were observed between the lower rates (60 and 80%) and the control N rate (100%). The BR rates were significantly higher by 10 to 13% in 120% N fertilization when compared with 60 and 80% of N fertilization applied over seven consecutive years. In contrast, the qCO_2_ was significantly lower, with the highest N fertilization (140%) compared to the others. In other words, the qCO_2_ decreased by 9% over time.

**Table 1:** Impact of nitrogen fertilization on soil depth distribution of microbial biomass, metabolic quotient, basal and specific maintenance respiration rates, and potentially mineralizable carbon concentration.

N fertilization (%)	Depth (cm)	MBC (mg/kg)	BR (mg/kg/d)	qCO_2_ (μg/mg/MBC/d)
60	0–15	176x^€^	13.8x	79y
	15–30	115.3y	13.3x	123x
	Mean	145.7b^≠^	13.5c	101a
80	0–15	193x	14.6x	76y
	15–30	130.3y	13.9x	119x
	Mean	161.7ab	14.2bc	98ab
100	0–15	192.5x	15.1x	86y
	15–30	135.3y	14.6x	122x
	Mean	163.9ab	14.8ab	104a
120	0–15	196.4x	15.7x	88y
	15–30	147.3y	14.9x	108x
	Mean	171.9a	15.3a	98ab
140	0–15	201.9x	15.4x	78y
	15–30	141.3y	14.7x	105x
	Mean	171.6a	15.1ab	92b

^≠^ Means separated by same lower-case letter (a, b, and c) under each column were not significantly different at p>0.05 among N fertilization rates.

^€^ Means separated by same lower-case letter (x and y) under each column were not significantly different at p>0.05 between soil depths at each N fertilization.

Results showed that the concentrations of TOC, POC, and POXC were significantly impacted by N fertilization (Table 2). While the TOC concentration was significantly higher by 10% when N was applied at 140% than that at 60% of the standard rates, the POC concentration was significantly higher by 46, 41, 21, and 10% in 140% with respect to 60, 80, 100, and 120% of the N fertilization, respectively. Likewise, the POXC concentration was significantly higher by 10 and 5% in 140% N fertilization with respect to 60 and 80% of N fertilization.

**Table 2:** Impact of nitrogen fertilization on soil depth distribution of total organic carbon, particulate organic matter, particulate organic carbon, and permanganate oxidizable carbon concentration.

N fertilization (%)	Depth (cm)	TOC (%)	POC (mg/kg)	POXC (mg/kg)
60	0–15	1.36x^€^	23.9x	463x
	15–30	1.26x	11.6y	428.6y
	Mean	1.3b^≠^	17.7d	445.8b
80	0–15	1.37x	23.6x	507.9
	15–30	1.27x	12.9y	421.5
	Mean	1.32b	18.3d	464.7b
100	0–15	1.41x	26.4x	525
	15–30	1.31x	16.5y	423.8
	Mean	1.36ab	21.4c	474.4ab
120	0–15	1.46x	27.6x	549.2
	15–30	1.33y	19.4y	408.1
	Mean	1.4ab	23.5b	478.6ab
140	0–15	1.49x	28.9x	555.3
	15–30	1.37y	22.9y	429.7
	Mean	1.43a	25.9a	492.5a

^≠^ Means separated by same lower-case letter (a, b, and c) under each column were not significantly different at p>0.05 among N fertilization rates.

^€^ Means separated by same lower-case letter (x and y) under each column were not significantly different at p>0.05 between soil depths.

When averaged across N fertilization rates, the MBC ranged from 176 to 202mg/kg in the 0–15 cm and 115 to 141 mg/kg in the 15–30 cm depths. In contrast, the qCO_2_ ranged from 76 to 88 μg CO_2_/mg MBC/d in the upper depth compared to 105 to 123 μg CO_2_/mg MBC/d in the lower depth, suggesting inefficient C metabolism. The BR showed a similar trend like MBC, and like the qCO_2_ for both depths. When averaged across N fertilization rates, the TOC, POC, and POXC concentration decreased significantly with depth.

When factored ρb to calculate C stocks, the depth distribution of TOC, POC, and POXC pools were variably impacted by N fertilization rates (Table 3). The TOC stock was significantly higher by 8.9% when N was applied at 140% than that at 60%, whereas the prior three rates were statistically alike to each other. A similar response of N fertilization was observed on POC stocks. However, the MBC stock was significantly higher at both 120 and 140% compared to the lowest rate (60%) of N fertilization, and the impact on the MBC stock was more pronounced (increased by 17%) when N was applied at 140% than at 60%. While the ρb increased, the MBC, POC, and POXC stocks consistently decreased with depth (Table 3).

**Table 3:** Impact of nitrogen fertilization on soil bulk density and depth distribution of total organic carbon, particulate organic matter, particulate organic carbon, and permanganate oxidizable carbon stocks.

N fertilization (%)	Depth (cm)	ρb (g/cm^3^)	TOC (Mg/ha)	POC (kg/ha)	POXC (Mg/ha)	MBC (Mg/ha)
60	0–15	1.29x^€^	26.4x	46.3x	0.9x	0.34x
	15–30	1.44y	27.2x	25.0y	0.93x	0.25y
	Mean	1.37a^≠^	26.8b	35.7d	0.91a	0.30b
80	0–15	1.29y	26.7x	45.8x	0.99x	0.38x
	15–30	1.44x	27.6x	27.9y	0.91x	0.28y
	Mean	1.37a	27.1ab	36.9d	0.95a	0.33ab
100	0–15	1.28y	27.1x	50.8x	1.01x	0.37x
	15–30	1.43x	28.0x	35.3y	0.91x	0.29y
	Mean	1.36a	27.6ab	43.1c	0.96a	0.33ab
120	0–15	1.29y	28.3x	53.4x	1.07x	0.38x
	15–30	1.43x	28.6x	41.7y	0.88y	0.32y
	Mean	1.47a	28.5ab	47.5b	0.98a	0.35a
140	0–15	1.3y	29x	56.2x	1.08x	0.39x
	15–30	1.44x	29.5x	49.5y	0.93y	0.31y
	Mean	1.37a	29.2a	52.9a	1.01a	0.35a

^≠^ Means separated by same lower-case letter (a, b, and c) under each column were not significantly different at p>0.05 among N fertilization rates.

^€^ Means separated by same lower-case letter (x and y) under each column were not significantly different at p>0.05 between soil depths.

### Nitrogen fertilization impact on soil carbon stratification and lability

While the N fertilization had a significant variable impact on POC, MBC, and POXC stratification, it did not affect TOC (Table 4). The POC stratification was higher (by 39 to 45%) at both 120 and 140% N fertilization followed by a 27% increase at 100% N fertilized when compared to the lowest rate (60% N fertilization). In contrast, the MBC stratification was higher (by 12 to 23%) at 80, 100, 120, and 140% N fertilization than at 60% N fertilization, where the 80, 100, 120, and 140% N fertilization rates were statistically alike. The POXC did not impact consistently by N fertilization, except by 140% N fertilization, which was significantly higher (by 11%) than that of the lowest N rate. The POC, MBC, and POXC stratification increased significantly with depth.

**Table 4:** Impact of nitrogen fertilization on stratification of total organic carbon, particulate organic matter, particulate organic carbon, and permanganate oxidizable carbon at different depths of soil.

N fertilization (%)	Depth (cm)	TOC	POC	POXC	MBC
60	0–15	1.07x^€^	2.17x	1.08x	1.53x
	15–30	1.00x	1.00y	1.00x	1.00y
	Mean	1.04a^≠^	1.58c	1.04b	1.26b
80	0–15	1.09x	2.13x	1.19x	1.69x
	15–30	1.01x	1.24y	0.99y	1.14y
	Mean	1.05a	1.68c	1.09ab	1.42a
100	0–15	1.11x	2.44x	1.23x	1.67x
	15–30	1.03x	1.56y	0.99y	1.17y
	Mean	1.07a	2.00b	1.12ab	1.42a
120	0–15	1.16x	2.51x	1.28x	1.70x
	15–30	1.05x	1.89y	0.95y	1.28y
	Mean	1.11a	2.20a	1.12ab	1.49a
140	0–15	1.18x	2.46x	1.30x	1.76x
	15–30	1.08x	2.14y	0.99y	1.22y
	Mean	1.13a	2.30a	1.15a	1.49a

^≠^ Means separated by same lower-case letter (a, b, and c) under each column were not significantly different at p>0.05 among N fertilization rates.

^€^ Means separated by same lower-case letter (x and y) under each column were not significantly different at p>0.05 between soil depths.

The CPI, as an indicator of TOC accumulation, was variably affected by N fertilization (Table 5). The CPI significantly increased by 10% at 140% N fertilization compared to the control, but other rates were statistically similar. The CL_i_, as a measure of TOC lability in response to management practices, was not affected consistently by N fertilization. The CL_i_ of POC was only affected by 100, 120, and 140% N fertilization when compared to the lower rates (60 and 80%) than the control; however, the latter rates did not vary among themselves. In contrast, the CMI, as a composite indicator of change in C accumulation and quality, responded positively to N fertilization. The CMI values of POC, MBC, and POXC increased with increasing rates of N fertilization. Both 120 and 140% N fertilization significantly increased the CMI values, and the response was more pronounced on POC than MBC and POXC when compared to their respective controls (100%). While the depth distribution of CPI did not vary, the CMI values of POC, MBC, and POXC consistently decreased with depth.

**Table 5:** Impact of nitrogen fertilization on soil depth distribution of carbon pool index, carbon liability index, and carbon management index.

N fertilization (%)	Depth (cm)	CPI	POXC		MBC		POC	
			CLi	CMI	CLi	CMI	CLi	CMI
60	0–15	1.20x^€^	1.20x	56.8x	1.08x	63.9x	1.14x	63.5x
	15–30	1.20x	0.99y	45.9y	0.77y	45.6y	0.61y	33.6y
	Mean	1.20b^≠^	1.10a	52.2b	0.93a	54.75b	0.88c	48.6c
80	0–15	1.32x	1.30x	61.9x	1.17x	69.9x	1.14x	62.6x
	15–30	1.32x	0.95y	45.3y	0.89y	51.8y	0.69y	37.7y
	Mean	1.32b	1.13a	53.6ab	1.03a	60.8a	0.92c	50.2c
100	0–15	1.34x	1.34x	64.2x	1.14x	70.2x	1.25x	70.5x
	15–30	0.92x	0.92y	45.4y	0.83y	53.5y	0.84y	48.2y
	Mean	1.13ab	1.13a	54.8ab	0.99a	54.9a	1.05a	59.4ab
120	0–15	1.34x	1.34x	66.6x	1.09x	71.3x	1.24x	73.1x
	15–30	0.90x	0.90y	43.6y	0.94x	58.5y	0.97y	56.9y
	Mean	1.12ab	1.12a	55.1ab	1.02a	64.9a	1.11a	65.0ab
140	0–15	1.32x	1.32x	67.8x	1.14x	78.8x	1.28x	76.3x
	15–30	0.89x	0.9y	45.7y	0.87y	55.9y	1.12y	66.7y
	Mean	1.10a	1.11a	56.8a	1.00a	67.4a	1.20a	71.5a

^≠^ Means separated by same lower-case letter (a, b, and c) under each column were not significantly different at p>0.05 among N fertilization rates.

^€^ Means separated by same lower-case letter (x and y) under each column were not significantly different at p>0.05 between soil depths.

### Relationship among soil organic carbon pools

**Fig 1:**
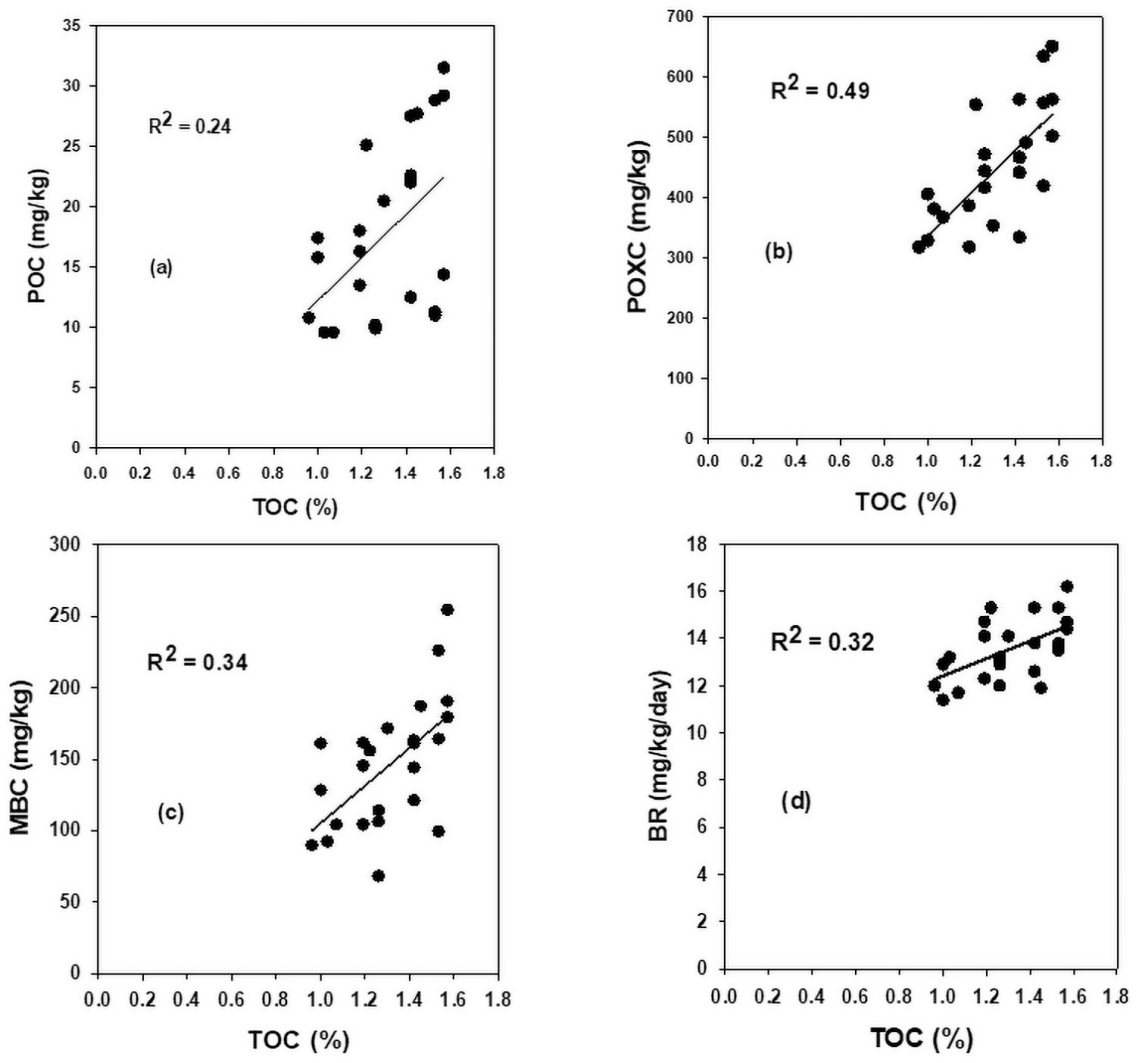
Relationship between (a) total organic carbon with particulate organic carbon (b) total organic carbon with permanganate oxidizable (c) total organic carbon with microbial biomass carbon, and (d) carbon total organic carbon with basal respiration rates, averaged across data.

**Fig 2:**
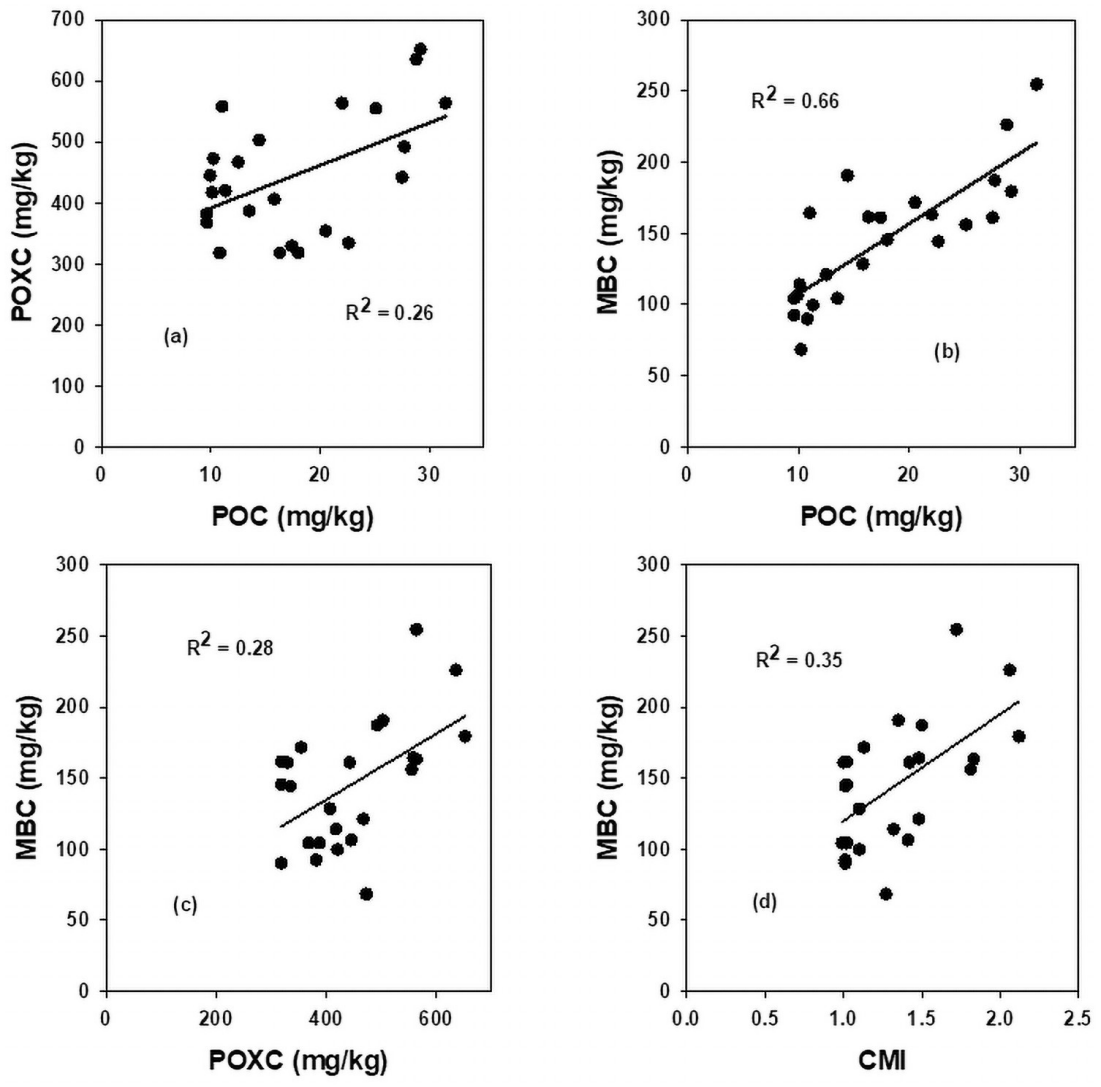
Relationship between (a) particulate organic carbon with permanganate oxidizable (b) microbial biomass carbon with particulate organic carbon, (c) microbial biomass carbon with permanganate oxidizable carbon, and (d) microbial biomass carbon with carbon management index, averaged across data.

**Fig 3:**
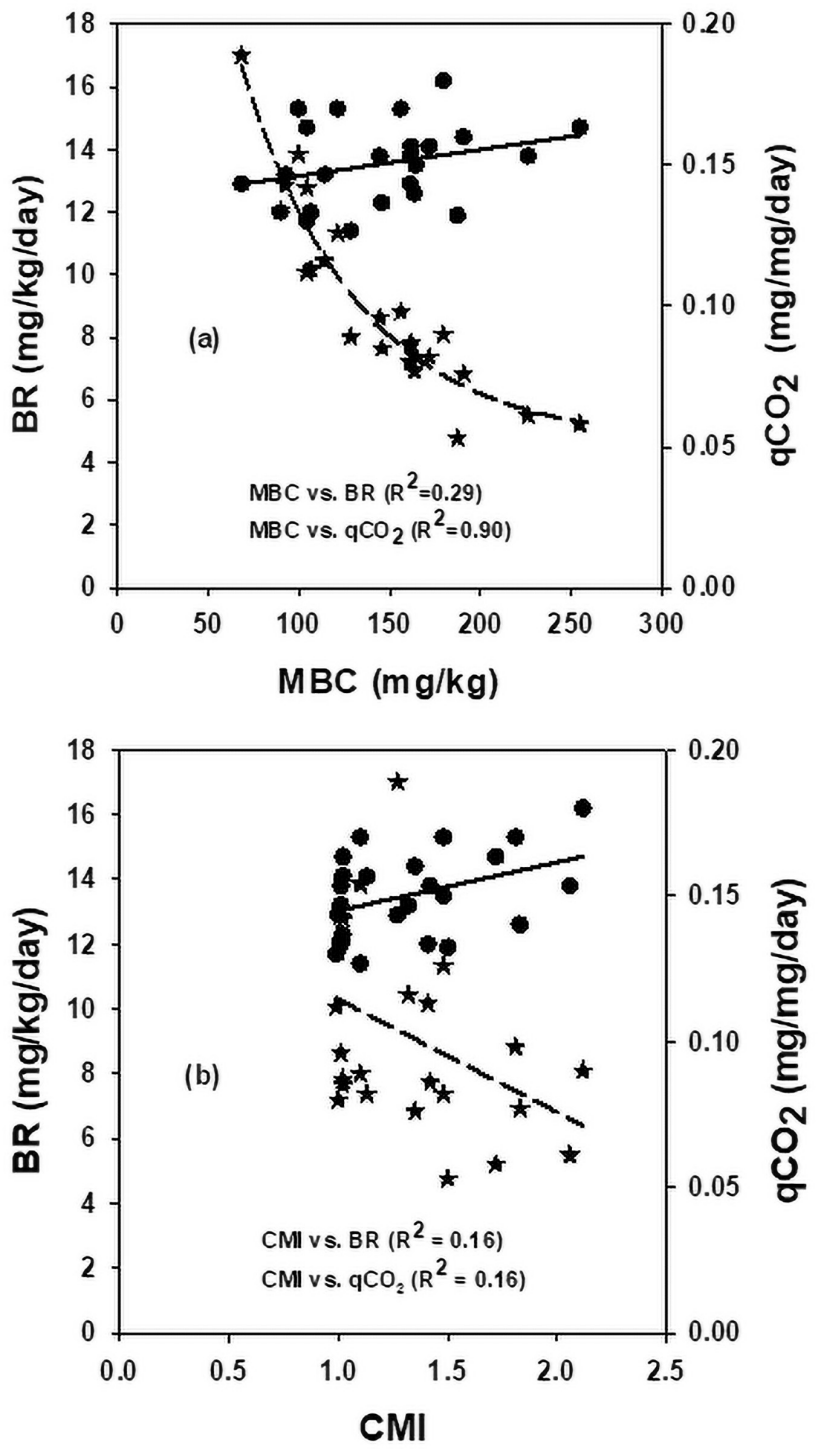
Relationship of (a) microbial biomass carbon with basal respiration and specific maintenance rates, and (b) carbon management index with basal respiration and specific maintenance rates, averaged across data.

Results showed that TOC pools variably responded among themselves to the impact of N fertilization (Figs 1–3). The TOC linearly accounted for 24, 49, 34 and 32% variability in POC, POXC, MBC and BR respectively (Fig 1). The MBC significantly varied for 66, 28 and 35% variability in POC, POXC, and CMI, respectively (Fig 2b–2d), and the POC significantly accounted for 26% of the variability in POXC (Fig 2a). Likewise, increasing MBC concentration, as influenced by N fertilization, linearly and moderately increased the BR rates by accounting for 29% of its variability. In contrast, the qCO_2_, as a measure of microbial catabolism, significantly (R^2^ = 0.90) decreased to a plateau (Fig 3a). In contrast, the CMI, showed a non-linear moderate relationship (R^2^ = 0.16) with BR rates with an associated decrease in qCO_2_ by accounting for 16% of its variability (Fig 3b).

## Discussion

### Nitrogen fertilization impact on soil organic pools

A significant increase in MBC was due to the impact of N fertilization on soil-crop ecosystems, a plausible cause of a more biologically compatible ecosystem for regulating SOM decomposition and other biochemical functions. N fertilization is expected to reduce soil C: N, which becomes close to microbial C: N over time and favors biological efficiency for SOM decomposition, thereby, resulting in an increase of MBC concentration and the size of the biologically labile pool (qR) of TOC. A greater availability of labile C is expected to provide food and energy substrates to support biodiversity and efficiency, resulting in higher MBC content [33]. The activity, size, and diversity of microbes depend on the quantity and quality of SOM, as influenced by management practices [34]. It is reported that cropping diversity synergistically affects soil microbiome with N fertilization by release root exudates, and consequently increased nutrient availability to produce more and diverse crop biomass [35, 36]. The increased MBC content was due to efficient turnover of root exudates and biomass produced in response to the impact of N fertilization. In other words, N fertilization not only required for high crop growth, but also for synthesis of root exudates for soil microbes.

While N fertilization increased the MBC content, it also increased BR rates, which may be due to the greater availability and utilization of TOC, either from SOM decomposition or root exudation. It is expected that N fertilization not only increased the decomposition of crop residue and native SOM within rhizosphere, but may have changed the utilization of TOC via changes in the microbial community. Our results are in line with other studies that reported N fertilization has been shown to increase biological activity and soil respiration [10, 37, 38]. However, a significant decrease in qCO_2_ with N fertilization was most likely influenced by microbial diversity and anabolism, which is expected to improve the energy-use efficiency of microbes [24, 39, 40]. It is reported that the N amendments support microbial diversity by enlarging the labile pool of TOC to fuel soil biology and their activities [41]. The changes in the MBC content can be driven by C availability (lability) upon the decomposition of TOC [42–44]. A relatively small, but labile pool of TOC was more closely related to the biological C pool than was the bulk TOC content [45, 46]. It is also expected that the impact of N fertilization increased the C availability by reducing both catabolism and progressive nutrient deficiency, thereby increasing the size and diversity of MBC pool and their efficiency.

Our results showed that N fertilization increased the size of the labile pool of TOC were consistent with the results of previous studies [47, 48]. The increase in labile C pool was partly attributed to the direct effect of N fertilization on crop biomass production and the chemistry of crop residue diversity. The proportion of recalcitrant C was reportedly lower (~ 63%) due to an increase in the size of labile pool of TOC under rice-wheat cropping systems [49]. It is reported that the TOC content increased in fertilized plots when compared to the control due to substantial amount of C added via the root and shoot biomass [50]. An increase in TOC by 10% with N fertilization, which is due to crop shoot, root, and rhizodeposition, observed in our studies agrees with previous results [51, 52]. However, a consistent and rapid change in the content of labile pools of TOC, such as POC, MBC, and POXC, were significantly affected by N fertilization when compared with the bulk TOC content [53]. Long-term N fertilization added more C input and transformation into both labile, but physically protected C in soil aggregates [54–56]. The C protected as POC within aggregates is an important source of labile C and energy for soil microbes and is comprised of fragmented and partially decomposed crop residues and microbial cell (fungal hyphae) and their metabolites [12, 57]. The POC, in return, acts as a cementing material helping to develop soil aggregate formation [58]. This was possible for the higher macroaggregates formation and occlusion of POC therein against microbial decomposition [57, 59]. The increase in POC accumulation was mainly due to the increase of plant residues as well as microbial and micro-faunal debris, including fungal hyphae and spores. Similar results were also reported by previous authors [60].

A significant variation in POXC content impacted by N fertilization was due to N stoichiometrically linked with C in SOM [61], therefore an increase in N invariably increases the TOC including the POXC content. The POXC is one of the labile (biochemically active) forms of TOC that contributes greatly to the soil quality functions and diversity of the soil food web and has been shown to be sensitive to crop-soil management practices [19, 27, 40]. While Jokela et al. [62] reported a greater sensitivity of POXC to management systems that was reflected in soil microbial and physical properties, others reported that POXC, as a measure of soil health, was capable of detecting management-induced changes in the labile C pool of soil [19, 27, 40, 63–66].

### Nitrogen fertilization impact on soil carbon stratification and lability

Significant stratification of TOC pools, especially POC, MBC, and POXC, was due to greater surface accumulation (at 0–15 cm depth) of a diverse quality of shoots and roots in a wheat-mungbean-rice cropping system under seasonal wetting-drying conditions. It is reported that carbon and post-applied nutrient stratification is a common phenomenon due to surface deposition and high affinity for complexation with soil reactive components [67, 68] including clay minerals, Ca, Mg, Fe, Al, and Mn and their consequent limited mobility in seasonally puddled tropical soils.

Long-term N fertilization is expected to produce higher crop biomass (both shoot and root) followed by surface accumulation and slower decomposition on partially wet soils, especially under transplanted rice and no-till wheat and mungbean cultural practices. The slower decomposition of crop residues is primarily because of partially cooler, wet, and anaerobic soil conditions and, consequently, the transformation and partition of crop-derived C into TOC pools by slow humification process was expected to be more biologically mediated. Likewise, biological activities associated with the decomposition of crop residues, including roots, are slower and incomplete due to O_2_ deficiency and lower soil-water temperatures [69]. In partially wet soil, continuing aerobic microbial respiration rapidly depletes dissolved O_2_ concentration and, consequently, anaerobic conditions develop because of restricted O_2_ diffusion through water, which is about 10,000 times slower than through the air. Martin and Holding [70] suggested that low soil-water temperature under partially wet soil conditions is one of the factors responsible for slower decomposition of crop residues and native SOM. In partially wet soil conditions, a portion of radiant energy is lost in evaporation at the water-soil interface, and this together with the high specific heat of water compared to soil minerals, suggesting partially wet and no-till soils warmed up slowly and, consequently, are responsible for a slower and incomplete decomposition of SOM [69]. Accordingly, the TOC pools, especially POC, MBC, and POXC, stratified at the surface soil due to the slow translocation downward and a lack of frequent incorporation of the crop residues into the deeper soil depths. Our results were collaborated with the results of other studies that reported on stratification of TOC pools [71, 72]. However, a greater stratification of labile pools of TOC, such as POC, POXC, and MBC, suggested that management practices involving N optimization is expected to improve the soil quality over time.

Significantly higher CL_i_ and CMI values observed in the N-fertilized soils are related to both the amount and quality of TOC accumulated over time, thus modifying the size of the labile C pools. Therefore, the CMI can be used as a sensitive indicator to monitor differences in TOC quality in response to management practices. Our results agree with other studies that reported that the impact of chemical fertilization significantly increased the CL_i_ and CMI values relative to the control [40, 53, 65, 73]. Soils with higher CL_i_ and CMI values are considered to be better managed.

### Relationships among soil carbon pools

A significant relationship of MBC with BR and qCO_2_, as influenced by N fertilization, suggested higher biological activities with improved C-use efficiency (qCO_2_), i.e., microbes, are allocating more C energy for cell growth with a significant decrease in C loss (energy efficiency) to carryout soil quality functions [24]. N-balanced soil is expected to be less stressed due to a significant decrease in the qCO_2_, and therefore, could see subsequent increases in POC, MBC, POXC, CL_i_, and CMI when compared to the control. Similarly, an increase in CMI associated with a non-linear increase in BR, but significant decrease in qCO_2_, implies a higher C-use efficiency by microbes with N fertilization. A significant relationship of TOC with MBC, POC, and POXC suggested that TOC, a composite of all C pools, regulates C partition in different gradients of lability to carryout soil quality properties and processes, or vice-versa [27, 40]. Moreover, the relationship of TOC with CMI implied that a small change in TOC accumulation is expected to affect TOC quality by partitioning in labile pools.

A linear relationship of MBC with POC and POXC suggested that MBC, under subtropical agroecosystems, utilizes both POC and POXC as labile C-substrates and energy sources to survive and carryout soil functions associated with crop production. A significant relationship between MBC and POXC suggests that soil microbes are part of labile C and contain a substantial amount of POXC in their cells, which is not surprising as POXC was measured on air-dried soil, which would have also lysed the microbial cells [65]. The significant positive relationship between MBC and POC affirms that both partially decomposed C and microbial metabolites are one of the cementing agents to form soil aggregates and physically protected itself within macroaggregates [74]. Likewise, the close relationship of MBC with CMI implied that microbial biomass and their activities with changes in C availability and quality, as influenced by N fertilization. An increase in MBC could be ascribed to high biological activity where substrates such as POC and POXC regulate the bioavailability of C in soil ecosystems [75]. A close relationship of POC with POXC indicated that both are complementary to each other as a measure of labile C pool of TOC.

## Conclusions

N fertilization at 120 to 140% over a period of seven years markedly decreased C loss (qCO_2_) via improving anabolism with an associated increase in the size of labile pool of TOC, especially POC, MBC, and POXC contents when compared to the control (100% N) under wheat-mungbean-rice in subtropical soil and climatic conditions. Increased POC, MBC, and POXC contents translated into stratification. A significant correlation among soil biological properties and labile pools of TOC suggested that higher N fertilization rates (120 to 140%) than the current recommended rate (100%) is expected to improve soil properties associated with improved soil quality. Therefore, optimization of N fertilization at 120% would be a suitable management strategy to maintain and/or improve soil quality in tropical rice-based agroecosystems.

## Supporting information

S1 Data (XLSX)
